# Stage IV colon cancer patients without DENND2D expression benefit more from neoadjuvant chemotherapy

**DOI:** 10.1038/s41419-022-04885-8

**Published:** 2022-05-06

**Authors:** Wen-juan Ma, Yukun Chen, Jian-hong Peng, Chaoming Tang, Ling Zhang, Min Liu, Shanshan Hu, Haineng Xu, Hua Tan, Yangkui Gu, Zhi-zhong Pan, Gong Chen, Zhong-guo Zhou, Rong-xin Zhang

**Affiliations:** 1 State Key Laboratory of Oncology in South China, Guangzhou, 510060 Guangdong Province People’s Republic of China; 2 Collaborative Innovation Center of Cancer Medicine, Guangzhou, 510060 Guangdong Province People’s Republic of China; 3 Intensive Care Unit Department, Sun Yat-Sen University Cancer Centre, Guangzhou, 510060 Guangdong Province People’s Republic of China; 4 Zhongshan School of Medicine, Sun Yat-Sen University, No. 74, Zhongshan Rd. 2, Guangzhou, 510080 Guangdong Province People’s Republic of China; 5 Department of Colorectal Surgery, Sun Yat-Sen University Cancer Centre, Guangzhou, 510060 Guangdong Province People’s Republic of China; 6 The Sixth Affiliated Hospital of Guangzhou Medical University, Qingyuan People’s Hospital, QingYuan, Guangdong Province People’s Republic of China; 7 Department of Radiology, Sun Yat-Sen University Cancer Center, Guangzhou, Guangdong Province People’s Republic of China; 8 Department of Ultrasound, Sun Yat-Sen University Cancer Center, Guangzhou, Guangdong Province People’s Republic of China; 9 Department of Statistics, Rutgers University, New Brunswick, NJ 08854 USA; 10 Ovarian Cancer Research Center, Division of Gynecology Oncology, Department of Obstetrics and Gynecology, University of Pennsylvania, Philadelphia, PA 19104 USA; 11 School of Biomedical Informatics, The University of Texas Health Science Center at Houston, Houston, TX 77030 USA; 12 Intervention Department, Sun Yat-Sen University Cancer Center, Guangzhou, Guangdong Province People’s Republic of China; 13 Department of Hepatobiliary Oncology, Sun Yat-Sen University Cancer Center, Guangzhou, Guangdong Province People’s Republic of China

**Keywords:** Colon cancer, Tumour biomarkers

## Abstract

According to the EPOC study, chemotherapy could improve 5-year disease-free survival of stage IV colon cancer patients by 8.1%. However, more molecular biomarkers are required to identify patients who need neoadjuvant chemotherapy. DENND2D expression was evaluated by immunohistochemistry in 181 stage IV colon cancer patients. The prognosis was better for patients with DENND2D expression than patients without DENND2D expression (5-year overall survival [OS]: 42% vs. 12%, *p* = 0.038; 5-year disease-free survival: 20% vs. 10%, *p* = 0.001). Subgroup analysis of the DENND2D-negative group showed that patients treated with neoadjuvant chemotherapy achieved longer OS than patients without neoadjuvant chemotherapy (RR = 0.179; 95% CI = 0.054–0.598; *p* = 0.003). DENND2D suppressed CRC proliferation in vitro and in vivo. Downregulation of DENND2D also promoted metastasis to distant organs in vivo. Mechanistically, DENND2D suppressed the MAPK pathway in CRC. Colon cancer patients who were DENND2D negative always showed a worse prognosis and were more likely to benefit from neoadjuvant chemotherapy. DENND2D may be a new prognostic factor and a predictor of the need for neoadjuvant chemotherapy in stage IV colon cancer.

## Introduction

Colon cancer is the leading cause of cancer-related mortality worldwide [1]. Surgical resection with or without adjuvant chemotherapy remains the standard treatment for nonmetastatic colorectal cancer (CRC) [2]. The survival of nonmetastatic CRC patients has improved with the development of multiple therapeutic strategies, including surgery, chemotherapy, and radiotherapy. However, improving the outcome of stage IV CRC remains a challenge for oncologists. Approximately 50–60% of patients diagnosed with CRC develop distant metastases [3]. Based on the report that almost half of colon cancer patients have liver metastasis at autopsy, liver metastases are the leading cause of death in CRC patients [4].

The median 5-year survival rate of colon cancer patients with liver metastasis (CCLM) could reach 38% if R0 resection could be performed [5]. The 5-year overall survival (OS) was as high as 71% following surgical resection for patients with a single lesion of liver metastasis [6–8]. According to the results of the EPOC study, systemic chemotherapy could improve disease-free survival (DFS) by 8.1% in CRC patients with liver metastasis, and chemotherapy was recommended for all patients with liver metastasis [9]. Whether CCLM should receive neoadjuvant chemotherapy remains debatable. CCLM patients with a poor prognosis could benefit more from neoadjuvant chemotherapy because micrometastatic disease could be treated earlier, and palliative surgery could be avoided for those with early disease progression [10]. However, no standard approach is available for these groups of patients. Especially for initially resectable CCLM patients, there is still controversy whether neoadjuvant chemotherapy should be administered. Most medical centers use the clinical risk score (CRS) to guide clinical practice [11]. For patients with high risk (CRS ≥3), neoadjuvant chemotherapy will be administered before surgical resection [11]. Patients with low risk (CRS ≤2) could receive surgical resection before adjuvant chemotherapy. However, CRS was created almost 20 years ago, and all variables are clinical parameters. Presently, more solid biomarkers are needed to help identify patients who might benefit from neoadjuvant chemotherapy.

Many studies have tried to molecularly characterize tumors [12–14]. Some studies have reported that RAS mutation is a negative prognostic factor for stage IV colon cancer patients [15–18]. Other studies have shown that RAS mutation status has no relationship with survival [18–21]. Therefore, the relationship between RAS mutation and survival remains unclear. BRAF mutation has a strong relationship with worse survival for stage IV colon cancer [21–23]. MSI-H colon cancer is a special subtype of colon cancer comprising a special subgroup of patients who could respond to immunotherapy [24]. Whether MSI could be a prognostic factor for stage IV colon cancer remains debatable [25, 26]. TP53 and PIK3CA gene mutations did not affect long-term outcomes [27, 28]. Many other potential molecular prognostic markers have been described, including POLE, POLD, HER2, NTRK, ALK, and ROS1 [29–32]; however, none are currently incorporated into routine clinical practice.

As a member of the DENND2 family, DENND2D consists of four subregions: the full DENN domain, the upstream DENN, the core DENN, and the downstream DENN. DENN/MADD (DENND) proteins represent a newly recognized class of membrane trafficking proteins that regulate Rab GTPases [33, 34]. DENND proteins play an important role as guanine nucleotide exchange factors for this GTPase and interact with Rab35 [35, 36]. However, the function of DENND2D in malignant tumors is still unclear. Previous studies investigated the function of DENND2D as a tumor-suppressor gene in gastric cancer (GC) [37, 38], lung cancer [39], hepatocellular carcinoma (HCC) [40], and bladder cancer [41]. DENND2D was reported to activate Rab pathways and function in intracellular signaling pathways [33, 34]. To our knowledge, no study has reported the relationship and regulatory mechanisms of DENND2D in CRC. In this study, we investigated the relationship among DENND2D expression, prognosis, and response to neoadjuvant chemotherapy in stage IV colon cancer patients.

## Materials and methods

### Patients and tissue samples

One hundred eighty-one samples were obtained from patients at Sun Yat-Sen University Cancer Center from May 1, 2003, to May 1, 2016. Informed consent was obtained from all the patients before tissue collection. All patients were diagnosed with initially resectable CRLM and received R0 or R1 surgical resection. All the patients were pathologically confirmed to have colon adenocarcinoma. And all the baseline information of patients was collected blindly, without knowing the expression level of DENND2D. Studies involving human tissue samples were carried out in accordance with the guidelines approved by the Ethics Committees of Sun Yat-Sen University Cancer Center (IRB NO. B2021–192–01).

### Cell culture

HCT116, HT29, and SW620 CRC cell lines were purchased from American Type Culture Collection (ATCC, Manassas, VA, USA) and cultured according to ATCC guidelines. HCT116 and HT29 cells were cultivated in McCoy’s 5A medium (KGM4892N; KeyGEN BioTECH), and SW620 cells were cultured in Leibovitz’s L-15 medium (KGM41300N; KeyGEN BioTECH) supplemented with 10% fetal bovine serum (P30–2302, Pan Biotech) and 1% penicillin-streptomycin (P4333; Sigma-Aldrich). The cells were maintained at 37 °C in 5% CO_2_. All the cell lines were authenticated by short tandem repeat analysis at the China Center for Type Culture Collection (Wuhan, China), and the absence of mycoplasma contamination was verified using a PCR detection kit (Shanghai Biothrive Sci. & Tech. Ltd.). The cells were frozen in liquid nitrogen and used for experiments in passages 3–10.

### SiRNA knockdown

Knockdown experiments were performed by transfecting 25 nM DENND2D siRNA (Sigma-Aldrich) into cells with Lipofectamine 3000 (L300075; Invitrogen). The RNA sequences were as follows: DENND2D: si-1, GGATGATTACGAGCCTATAAT; si-2, CCATTATGCTTCCTATATCAA; si-3, TTGGATCCCTGGTATTGATTT. Si-Ctrl is the Mission shRNA nontargeting Pool from Sigma-Aldrich. The cells were transfected with siRNAs for 72 h; siRNA suppression of target protein was validated by western blot analysis.

### Generation of stable cell lines

The full-length cDNA of human DENND2D was cloned into the pCDH-EF1α-MCS-T2A-Puro Cloning and Expression Lentivector (CD526A-1; System Bioscience) tagged with 3×Flag. The shRNA sequences targeting DENND2D (sh1 and sh2) and negative control shRNA (shNC) were inserted into the pLko.1 Cloning and Expression Lentivector (SHC001; Sigma-Aldrich), and the sequences were as follows: DENND2D: sh1, CCGGTTGGATCCCTGGTATTGATTTCTCGAGAAATCAATACCAGGGATCCAATTTTTG; sh2, CCGGCCATTATGCTTCCTATATCAACTCGAGTTGATATAGGAAGCATAATGGTTTTTTG. HEK293T cells were transfected using Lipofectamine 3000 with pSPAX2, pMD2. G and these recombinant plasmids. Stable overexpression (-vec, -DENND2D) or knockdown (-shNC, -sh1/2) cell lines were used after selection with puromycin (1–2 µg/ml; BS080A; Biosharp) for 7 days and verification by western blot analysis.

### Western blotting (WB)

Cells were lysed in lysis buffer (9803; Cell Signaling Technology) supplemented with a protease and phosphatase inhibitor cocktail (78442; Thermo Fisher Scientific), and the protein concentration was quantified using a BCA protein assay kit (KGP903; KeyGen Biotech). The protein samples were denatured at 95 °C for 10 min and separated by SDS-PAGE. PageRuler Prestained Protein Ladder (26616) (Thermo Fisher Scientific) was used as a size standard to indicate the molecular weight, and the proteins were transferred onto PVDF membranes (03010040001; Sigma-Aldrich), which were blocked with 5% milk in TBS and incubated overnight at 4 °C with specific antibodies against the following proteins: DENND2N (ab184799; Abcam); GAPDH (D16H11), phosphor-MEK1/2 (Ser221) (166F8), MEK1/2 (L38C12), phosphor-ERK1/2 (Thr202/Tyr204) (#9101), and ERK1/2 (20G11). Subsequently, the membranes were incubated with HRP-conjugated goat anti-rabbit IgG (#7074) or anti-mouse IgG (#7076P2) (CST). The signals were visualized using an enhanced chemiluminescence reagent (WP20005; Thermo Fisher Scientific) for immunoblotting. The proteins were analyzed using the ChemiDoc XRS system (Bio-Rad).

### Clonogenic assay

Cells were seeded into six-well plates at a density of 1 × 10^3^ cells per well and cultured for 10–14 days at 37 °C to allow colony formation. The colonies (containing more than 50 cells) were stained with 0.5% crystal violet and counted.

### CCK-8 assay

Cell viability was determined using a CCK-8 kit (HY-K0301; MCE). Briefly, cells (2500/well) were cultured in 96-well plates for 72 h. Ten microliter of CCK-8 (Dojindo Laboratories) was added to each well, and the cells were incubated for an additional 3 h at 37 °C. The absorbance was then measured at 450 nm using a scanning multiwell spectrophotometer (Thermo Scientific). Cells were treated with various concentrations of 5FU for 72 h. The 50% inhibitory concentration (IC_50_) value was calculated.

### Quantitative reverse transcription-PCR

Total RNA was isolated from cells and tissues using the TRIzolTM RNA Purification Kit (12183555; Invitrogen). cDNA was obtained by reverse transcription using the M-MLV Kit (M1705; Promega) for qRT-PCR according to the manufacturer’s instructions. iTaq SYBR Green Mix (720001564; Bio-Rad) was used for qRT-PCR gene expression analysis, which was performed on the Bio-Rad CFX platform. The relative DENND2D mRNA levels were normalized to the actin mRNA levels. The primer sequences were as follows:

DENND2D

forward: 5’-CACTGCTCTACCCCTTCAGC-3’;

reverse: 5’-TTTTTCATCACCAACCGACA-3’.

Actin

Forward: 5’-CGTGAAAAGATGACCCAGATCA-3’;

Reverse: 5’-CACAGCCTGGATGGCTACGT-3’.

### Nude mouse xenograft model, liver metastasis model, and in vivo analysis

The animal experiments were approved by the SYSUCC Institutional Animal Care and Usage Committee in accordance with the Animal Welfare and Rights in China. Female BALB/c nude mice (4–5 weeks; 15–18 g; SLRC Laboratory Animal Co., Shanghai, China) were divided into five groups with five mice each randomly and used to generate xenograft models by injecting HCT116 cells (shNC; DENND2D-knockdown-sh1/sh2 or Vector; DENND2D-overexpression) into the right flanks of the mice. Starting on the 7th day post transplantation, mouse xenografts were monitored every 3 days for tumor formation, and mice were sacrificed when the largest diameter reached 20 mm, and the tumors were resected and weighed. The tumor volume was calculated using the following formula: tumor volume = 0.52 × width^2^ × length. To study liver metastasis, we established a mouse liver metastasis model by injecting stably modified HCT116 cell lines into the spleen. We injected 5 × 10^6^ cells from each clone into the spleens of BALB/c nude mice. Three weeks later, all mice were sacrificed to examine the livers for metastases [42].

### Tissue microarrays and immunohistochemistry

We used a tissue array instrument (personal tissue arrayer, Beeche, USA) to convert the paraffin-embedded specimens of individual tumor and normal mucosa into construction [43]. Immunohistochemistry (IHC) was performed as previously reported [40]. Briefly, paraffin-embedded specimens were serially cut into three 4-μm-thick sections. One section was used for routine hematoxylin and eosin staining, while the other two sections were used for staining using the streptavidin peroxidase (SP) IHC method. The procedures were performed according to the manufacturer’s instructions for each reagent kit. After deparaffinization and rehydration, sections were washed three times in PBS and boiled in a high-pressure cooker for 2.5 min in EDTA buffer (pH 8.0) for antigen retrieval. Non-specific binding was blocked using 5% BSA, after which the sections were consecutively incubated with the primary antibody, secondary antibody, and enzyme-labeled SP. Finally, the sections were developed using 3,3’-diaminobenzidine and counterstained with hematoxylin. The stained sections were cleared, mounted, and examined under a microscope.

The primary antibody solution consisted of a rabbit anti-human DENND2D polyclonal antibody (1:200 dilution; HPA048642; Sigma-Aldrich, USA), p-ERK (1:200 dilution, #4370, Cell Signaling Technology, Inc.) in blocking buffer, or ki67 (Working Concentration; ZM-0167, Zhongshan Golden Bridge Bio-technology, Beijing, China), and it was incubated with the sections at 4 °C overnight in a humidified chamber. Blocking buffer without the primary antibody was used as a negative control.

Each slide was evaluated using the IHC scoring system used in our previous study [44]. If the conclusions of the two pathologists differed, a third pathologist independently evaluated each case and decided the final score.

### Gene expression data and statistical analyses

Gene expression data of each cancer were downloaded from the TCGA data portal (https://portal.gdc.cancer.gov/) (Supplementary Table 1). A total of 20,531 genes (protein coding and noncoding) were included in the TCGA Illumina HiSeq RNASeq V2 data. We used level 3 gene expression data, which were derived from the reads per kilobase of transcript per million reads mapped (RPKM). The gene expression values were logarithmically transformed (base 2) prior to further analysis. Gene expression was visualized with box plots by the R package ggplot2, version 3.3.5.

The clinical and follow-up data were analyzed using SPSS v19.0 and R language. *χ*^2^, continuity correction *χ*^2^, and Fisher’s exact tests were used to assess the patients’ baseline variables. The significance of the variables was tested using Kaplan–Meier, multivariate Cox regression, and logistic regression models. The variation of each group had been estimated properly and met the basic assumptions of the tests. OS was defined as the interval between surgical resection and death or the end of follow-up. DFS was defined as the interval between surgical resection and recurrence, metastasis, or the end of follow-up. Values of *p* < 0.05 indicated significant differences. All data in our study have been recorded at Sun Yat-Sen University Cancer Center for future reference (Number RDDB2021001606).

## Results

### DENND2D expression in TCGA database and TNMplot web tool

**Fig. 1:**
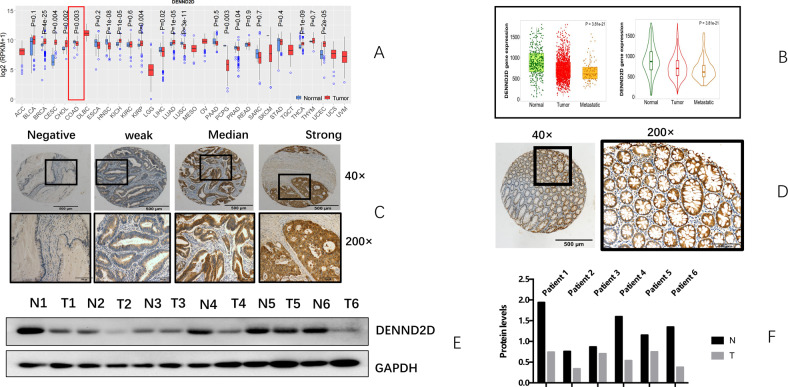
DENND2D is more highly expressed in adjacent normal tissues than colorectal cancer tissues, confirmed by online database and our clinical samples. **A** Expression level of DENND2D across 31 TCGA cancer types, in comparison with their normal controls if available. The middle line in the box is the median, the bottom and top of the box are the first and third quartiles, the whiskers extend to 1.5 IQR of the lower quartile and the upper quartile respectively, and the black solid circles represent outliers. *P* values were derived from two-sided Wilcoxon’s rank-sum test. **B** DENND2D gene expression among normal, tumor, and metastatic tissues in colorectal cancer patients obtained from the TNMplot.com web tool. **C** Protein expression of DENND2D in colon cancer tissues from 181 stage IV CRC patients. The level of DENND2D was classified as negative, weak, moderate, and strong. Protein expression of DENND2D in normal tissue (×40 and ×200). **D** Protein expression of DENND2D in normal tissues. **E**, **F** Western blot analysis of six pairs of tumor tissues and normal tissues from six colon cancer patients.

Different gene expression data of each cancer were downloaded from TCGA data portal (https://portal.gdc.cancer.gov/) (Supplementary Table). A total of 20,531 genes (protein coding and noncoding) were included in the TCGA Illumina HiSeq RNASeq V2 data (Supplementary Table). DENND2D was expressed at higher levels in normal tissue than in the tumor tissue in colon cancer (*p* = 0.003). Next, we confirmed our results using the TNMplot web tool [45] (Fig. 1B).

### General characteristics of the patients

From May 1, 2003, to May 1, 2016, 181 patients who were pathologically confirmed to have stage IV colon cancer were recruited from the Sun Yat-Sen University Cancer Center. All patients were initially deemed resectable as determined by two hepatic surgeons. In the end, 123 patients (67.9%) received R0 resection, and 39 patients (32.1%) received R1 resection. Among all the patients in this study, samples from 150 patients (82.1%) were DENND2D positive, and 31 (17.9%) were DENND2D negative. Forty-eight patients (26.5%) received neoadjuvant chemotherapy and 133 patients (73.5%) did not.

### DENND2D expression in colorectal cancer tissue and normal tissue

Compared with normal tissue, DENND2D expression was lower (Fig. 1A). DENND2D expression was examined in the tissues of 181 colon cancer patients and in normal tissues by IHC (Fig. 1C, D). The expression level of DENND2D was defined as negative, weak, moderate, and strong. According to the percentage of positive cells, the results were classified as follows: negative (negative was defined as no DENND2D expression) and positive (weak, moderate, and strong expressions were defined as positive). The classification of IHC expression levels was performed as previously described [46]. Six pairs of tumor tissue and normal colon tissue from six colon cancer patients were also used to evaluate DENND2D expression by WB (*p* = 0.01, Fig. 1E, F). We confirmed that DENND2D expression was stronger in normal tissue than in tumor tissue.

### DENND2D expression and survival

In total, 181 patients were followed up until December 1, 2020, and were included in the survival analysis. No other difference, in terms of characteristics including sex, age, and tumor location, was detected between the DENND2D-positive and DENND2D-negative groups (more details are provided in Table 1). The median follow-up period was 27 months. At the end of the follow-up time, 9 patients (29.0%) in the DENND2D-negative group were alive, while 69 patients (43.1%) in the DENND2D-positive group were alive.

**Table 1:** Clinicopathologic characteristics of stage IV CRC patients.

Characteristic	DENND2D negative (*n* = 31)	DENND2D positive (*n* = 150)	*p* value
Sex			0.222
Male	17 (54.8%)	100 (66.7)	
Female	14 (45.2%)	50 (33.3%)	
Age	55.55 (50.04–61.06)	55.93 (53.78–60.66)	0.888
Tumor location			0.676
Ascending colon	10 (32.3%)	43 (28.7%)	
Transverse colon	1 (3.2%)	6 (4.0%)	
Descending colon	4 (12.9%)	12 (8.0%)	
Sigmoid colon	9 (29.0%)	63 (42.0%)	
Rectum	7 (22.6%)	26 (17.3%)	
Surgery resection			0.526
R0	23 (74.2%)	119 (79.3%)	
R1	8 (25.8%)	31 (20.7%)	
CEA	9.78 (0.96–2586)	13.81 (0.35–4935)	0.181
CA199	13.99 (0.60–10,426.40)	26.77 (0.60–20,000)	0.176
Surgery procedure			0.584
Right hemicolectomy	12 (38.7%)	42 (28.0%)	
Transverse colectomy	0 (0%)	5 (3.3%)	
Left colectomy	3 (9.7%)	15 (10.0%)	
Sigmoidectomy	6 (19.4%)	35 (23.3%)	
Anterior resection	9 (29.0%)	51 (34.0%)	
Abdominal perineal resection	0 (0%)	1 (0.7%)	
Hartmann	1 (3.2%)	1 (0.7%)	
Neoadjuvant chemotherapy			0.728
None	9 (29.0%)	39 (26.0%)	
Yes	22 (71.0%)	111 (74.0%)	
Neoadjuvant chemotherapy			0.069
None	9 (29.0%)	39 (26.0%)	
FOLFIRI	2 (6.5%)	14 (9.3%)	
FOLFOX	6 (19.4%)	29 (19.3%)	
XELOX	12 (38.7%)	58 (38.7%)	
XELODA	0 (0%)	7 (4.7%)	
XELIRI	1 (3.1%)	0 (0%)	
XELOX + XELODA	0 (0%)	2 (1.3%)	
5FU/CF	1 (3.2%)	1 (0.7%)	

**Fig. 2:**
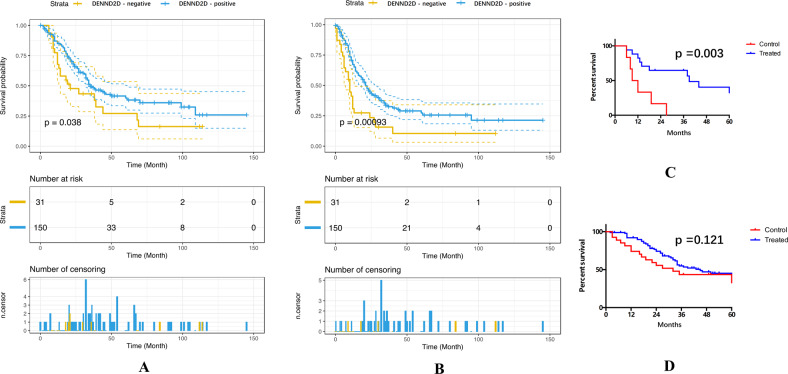
DENND2D-negative patients with R0 resction benefit more from neoadjuvant chemotherapy. **A** Overall survival of 181 stage IV colon cancer patients with 95% confidence intervals. **B** Disease-free survival of 181 stage IV colon cancer patients with 95% confidence intervals. **C** Comparison of the survival rate between patients who had received R0 resection with or without neoadjuvant chemotherapy in the DENND2D-negative group. **D** Comparison of the survival rate between patients who had received R0 resection with or without neoadjuvant chemotherapy in the DENND2D-positive group.

Univariate analysis revealed that DENND2D expression (RR = 0.611; 95% CI: 0.381–0.981; *p* = 0.038), CEA level (RR = 1.003; 95% CI: 1.000–1.004; *p* = 0.001), and neoadjuvant chemotherapy (RR = 0.615; 95% CI: 0.403–0.938; *p* = 0.024), R0 resection (RR = 0.542; 95% CI: 0.335–0.876; *p* = 0.012), N stage (RR = 0.419; 95% CI: 0.240–0.732; *p* = 0.001), and number of lymph nodes with metastasis (RR = 0.970; 95% CI: 0.944–0.997; *p* = 0.032) were strongly related to OS among colon cancer patients (Fig. 2A). In addition, DENND2D expression (RR = 0.493; 95% CI: 0.319–0.761; *p* = 0.00093), the CEA level (RR = 1.00; 95% CI: 1.000–1.001; *p* = 0.015), the CA19–9 level (RR = 1.00; 95% CI: 1.000–1.001; *p* = 0.001), number of lymph nodes with metastasis (RR = 0.967; 95% CI: 0.944–0.991; *p* = 0.008), and N stage (RR = 0.595; 95% CI: 0.369–0.961; *p* = 0.034) affected DFS (Fig. 2B). Other variables showed no relationship with the survival of colon cancer patients (additional details are presented in Table 2).

**Table 2:** Univariate and multivariate analyses of prognostic factors for disease-free survival and overall survival in 181 stage IV colorectal cancer patients.

	DFS				OS			
	Univariate		Multivariate		Univariate		Multivariate	
Variable	RR (95% CI)	*p*	RR (95% CI)	*p*	RR (95% CI)	*p*	RR (95% CI)	*p*
Sex	0.790 (0.549–1.138)	0.206			0.843 (0.564–1.262)	0.408		
Age	0.998 (0.985–1.010)	0.723			1.003 (0.988–1.017)	0.713		
T stage	0.912 (0.656–1.266)	0.580			1.415 (0.194–10.346)	0.732		
N stage	0.595 (0.369–0.961)	**0.034**	0.448 (0.269–0.747)	**0.002**	0.419 (0.240–0.732)	**0.001**	0.291 (0.160–0.528)	**0.001**
LN	0.967 (0.944–0.991)	**0.008**			0.970 (0.944–0.997)	**0.032**		
Ki67	1.005 (0.994–1.015)	0.379			1.005 (0.099–1.017)	0.369		
Positive LN	1.029 (0.981–1.079)	0.241			1.031 (0.979–1.085)	0.247		
R0 resection	0.766 (0.494–1.188)	0.233			0.542 (0.335–0.876)	**0.012**		
Tumor size	0.932 (0.833–1.043)	0.216			0.919 (0.806–1.049)	0.210		
Pathology	1.249 (1.009–1.545)	0.051	4.782 (1.293–17.68)	**0.019**	0.713 (0.224–2.268)	0.566	5.499 (0.582–51.953)	**0.001**
DENND2D expression	0.493 (0.319–0.761)	**0.001**	0.353 (0.210–0.595)	**0.001**	0.611 (0.381–0.981)	**0.041**	0.327 (0.207–0.517)	**0.001**
CEA	1.00 (1.000–1.001)	**0.015**			1.003 (1.000–1.004)	**0.001**		
CA 19–9	1.00 (1.000–1.001)	**0.001**			1.000 (1.000–1.001)	**0.001**		
Neoadjuvant chemotherapy	0.933 (0.627–1.387)	0.730			0.615 (0.403–0.938)	**0.024**		
Tumor location	0.935 (0.835–1.049)	0.251			1.399 (0.798–2.453)	0.241		

Bold values indicate statistically significant *p* values.

Cox multivariate analysis revealed that the significant prognostic factors for DFS were DENND2D expression (RR = 0.353; 95% CI = 0.210–0.595; *p* = 0.001), N stage (RR = 0.448; 95% CI = 0.269–0.747; *p* = 0.002) and pathology types (RR = 4.782; 95% CI = 1.293–17.68; *p* = 0.019). DENND2D expression (RR = 0.327; 95% CI = 0.207–0.517; *p* = 0.001), pathology types (RR = 5.499; 95% CI = 0.582–51.953; *p* = 0.001), N stage (RR = 0.291; 95% CI = 0.160–0.528; *p* = 0.001) were significant prognostic factors for OS (additional details are presented in Table 2).

### DENND2D was associated with the efficacy of neoadjuvant chemotherapy in the R0 resection group

Kaplan–Meier analysis was used to examine the relationship between neoadjuvant chemotherapy, DENND2D expression, and long-term survival in 123 patients who had received R0 surgical resection. In the DENND2D-negative group (including 23 patients), the patients who had received neoadjuvant chemotherapy achieved a longer median survival time than those without neoadjuvant chemotherapy (RR = 0.179; 95% CI = 0.054–0.598; *p* = 0.003; Fig. 2C). Regarding DENND2D-positive patients, neoadjuvant chemotherapy did not improve long-term survival significantly compared with those without neoadjuvant chemotherapy (RR = 0.659; 95% CI = 0.093–4.262; *p* = 0.121; Fig. 2D). For the patients who only received R1 resection, neoadjuvant therapy did not improve long-term survival regardless of whether DENND2D expression was positive or negative. The most significant difference was found in the DENND2D-positive group with neoadjuvant chemotherapy and the DENND2D-negative group without neoadjuvant chemotherapy (Supplementary Fig. 1).

### DENND2D suppressed CRC cell proliferation and metastasis in vitro and in vivo

**Fig. 3:**
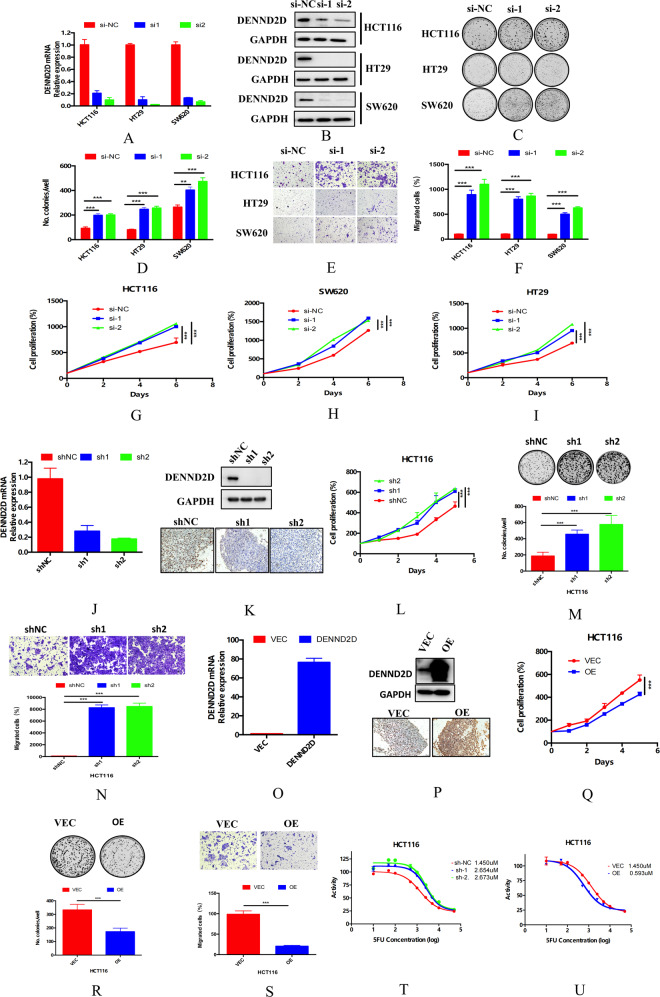
DENND2D suppressed tumor proliferation, migration and colony formation in vitro. **A**, **B** DENND2D was knockdown by siRNA in HCT116, HT29 and SW620 confirmed by qPCR and western blot. **C**, **D** CRC cell proliferation was promoted by DENND2D knockdown. **E**, **F** CRC cell migration was promoted by DENND2D knockdown. **G**–**I** CRC cell proliferation was promoted by DENND2D knockdown. **J**, **K** DENND2D was knocked down by shRNA in HCT116 confirmed by qPCR, western blot, and IHC. **L**, **M** CRC cell proliferation was promoted by DENND2D knockdown. **N** CRC cell migration was promoted by DENND2D knockdown. **O**, **P** DENND2D was overexpressed in CRC cells by shRNA confirmed by qPCR, western blot, and IHC. **Q**, **R** CRC cell proliferation was suppressed by DENND2D overexpression. **S** CRC cell migration was suppressed by DENND2D overexpression. **T**, **U** CRC cells are more sensitive to 5FU after DENND2D knockdown.

**Fig. 4:**
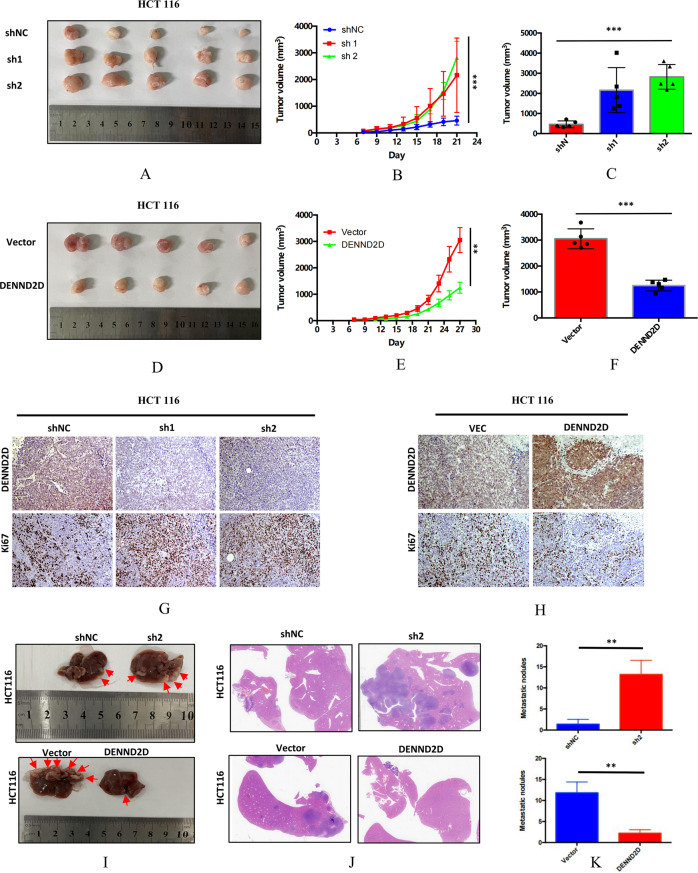
DENND2D suppressed CRC cell proliferation and metastasis in vivo. **A**–**C** Volumes of xenografts generated from HCT116-sh1/sh2 and HCT-shNC cells. **D**–**F** The volumes of xenografts generated from HCT116 and HCT overexpressing cells. **G**, **H** DENND2D and Ki67 expression in xenografts generated from HCT116-sh1/sh2, HCT-shNC, HCT116, and DENND2D-overexpressing cells. **I**–**K** Liver metastasis model generated by spleen injection of HCT116-sh2, HCT-shNC, HCT116, and DENND2D-overexpressing cells.

Compared with normal tissue, DENND2D was expressed at a lower level in CRC tissues, and patients with lower DENND2D expression were more likely to show a poor prognosis than those with higher DENND2D expression. To determine whether DENND2D is a tumor-suppressor gene for CRC, we investigated the relationship among DENND2D expression, CRC tumorigenesis, and progression. First, we knocked down DENND2D expression in the colon cancer cell lines HCT116, HT29, and SW480 (Fig. 3A) by siRNA and confirmed the results by western blot assays (Fig. 3B). To explore the function of DENND2D in CRC cell lines, colony formation, CCK-8, and migration assays were performed. DENND2D knockdown significantly promoted CRC cell proliferation and migration (Fig. 3C–F, G, I). According to the DENND2D expression level in common CRC cell lines (Supplementary Fig. 2A), we used shRNA to knock down DENND2D expression and established stable colon cell lines, which were confirmed by qPCR, WB, and IHC (Fig. 3J, K). Next, CCK-8, colony formation, and migration assays were performed. DENND2D knockdown significantly promoted CRC cell proliferation and migration (Fig. 3L–N). The same results were also obtained in the HT29 cell line (Supplementary Fig. 2B–F). We also overexpressed DENND2D in the HCT116 cell line, which was confirmed at both the transcriptional and protein levels (Fig. 3O, P). The CCK-8, colony formation, and migration assay results showed that DENND2D overexpression significantly suppressed CRC cell proliferation and migration (Fig. 3Q–S). The same results were also obtained in the RKO cell line (Supplementary Fig. 3A–E). By calculating the 50% inhibitory concentration (IC_50_) value, we noticed that HCT116 cells were less sensitive to 5FU after DENND2D knockdown, and DENND2D-overexpressing HCT116 cells were more sensitive to 5FU (Fig. 3T, U). In addition, the results were confirmed at both levels in HT29 and RKO cell lines (Supplementary Figs. 2G and 3F). Next, a xenograft model was used to test whether DENND2D could influence the growth of CRC in nude mice. DENND2D-silenced or DENND2D-overexpressing HCT116 cells were subcutaneously injected into the flanks of nude mice to establish a xenograft model (5 mice in each group). The volumes of xenografts derived from HCT116-sh1/sh2 cells were significantly larger than those from HCT116-shNC cells (Fig. 4A–C), whereas DENND2D overexpression significantly suppressed tumor growth (Fig. 4D–F). IHC of animal tumor samples confirmed that DENND2D was knocked down or overexpressed in stable cell lines (Fig. 4G, H). We established a mouse liver metastasis model by injecting HCT116 cell lines stably expressing shRNA or overexpressing DENND2D into the spleen. DENND2D-silenced HCT116 cells produced more metastatic lesions in the liver, and the DENND2D-overexpressing HCT116 group had fewer liver metastatic lesions (Fig. 4I–K). Collectively, these findings indicate that DENND2D suppresses CRC cell proliferation and metastasis in vitro and in vivo, regulating CRC tumorigenesis.

### DENND2D functions by suppressing the MAPK pathway in CRC tumorigenesis

**Fig. 5:**
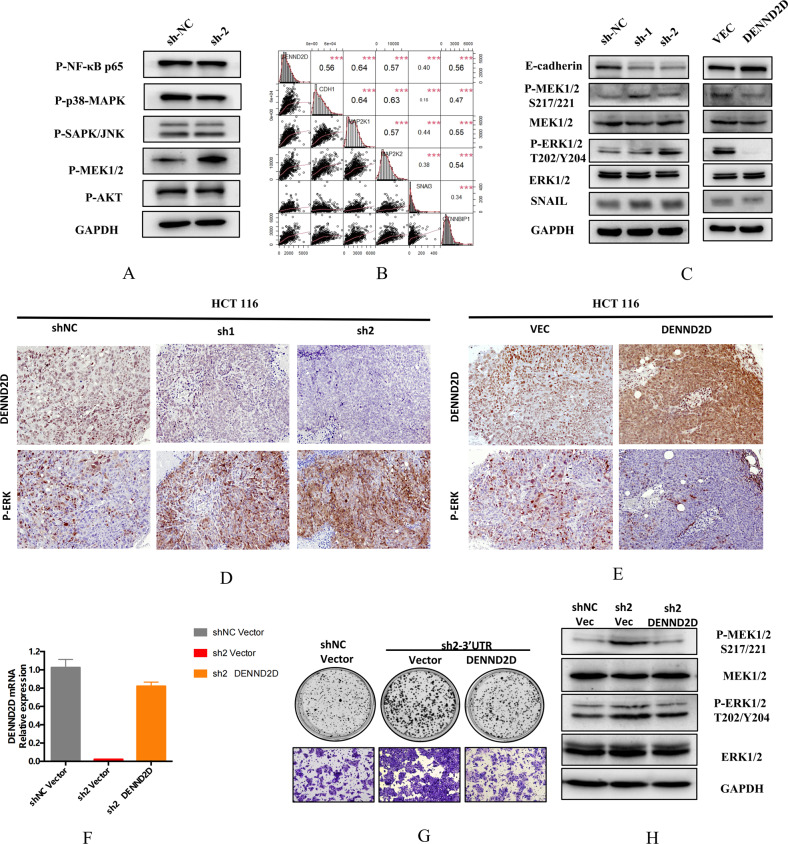
DENND2D suppressed CRC cell proliferation and metastasis in vitro by MAPK pathway. **A** Differential pathway-related protein expression between shNC and sh2 DENND2D cell lines. **B** The relationship between six key molecules using Pearson correlation analysis of the data from the TCGA database. **C** DENND2D overexpression or knockdown changed the levels of p-MET1/2 (Ser221) and p-ERK1/2 (Thr202/Tyr204) in HCT116 cells, as determined by WB assay. **D**, **E** DENND2D and pERK were expressed in xenografts generated from HCT116-sh1/sh2, HCT-shNC, HCT116, and DENND2D-overexpressing cells. **F**–**H** Restoring DENND2D expression in DENND2D-silenced CRC cells rescued the activity of the MAPK pathway.

To elucidate the molecular mechanisms by which DENND2D contributes to CRC tumorigenesis, we examined the expression of different possible pathway-related proteins between shNC HCT116 and sh2 HCT116 cells to identify possible signaling pathways involved. MEK was significantly upregulated after DENND2D was knocked down (Fig. 5A). We also confirmed the correlation of five related genes in the TCGA database (Fig. 5B). Therefore, we hypothesized that DENND2D functions by suppressing the MAPK pathway. WB assays revealed that DENND2D knockdown increased the levels of p-MEK1/2 (Ser221) and p-ERK1/2 (Thr202/Tyr204), whereas DENND2D overexpression resulted in decreased levels of p-MEK1/2 and p-ERK1/2 in HCT116 cells (Fig. 5C). We also confirmed our results in animal tumor samples. IHC demonstrated that DENND2D overexpression decreased the levels of p-ERK1/2 (Thr202/Tyr204), whereas DENND2D knockdown resulted in increased levels of p-ERK1/2 in HCT116 cells (Fig. 5D, E). To confirm the results, we restored DENND2D expression in DENND2D-silenced CRC cells by transiently transfecting a DENND2D expression plasmid and found that the re-expression of DENND2D decreased the activity of the MAPK pathway (Fig. 5F–H). In summary, DENND2D functions by suppressing the MAPK pathway in CRC tumorigenesis.

## Discussion

DENND2D, a regulator of Rab GTPases, is a member of the DENND2 family [36]. DENND2D plays a crucial role in cancer proliferation and metastasis. However, little is known concerning the relationship between DENND2D expression and colon cancer. In a non-small cell lung cancer cell line, DENND2D was identified as a tumor-suppressor gene. The same phenomenon was also observed in HCC [40], esophageal squamous cell carcinoma [47], and GC [48]. In our study, DENND2D acted as a tumor-suppressor gene. DENND2D expression was downregulated in CRC tissues compared with that in normal tissues in colon cancer samples from six stage IV patients. By knocking down DENND2D expression in HCT116, HT29, and SW480 cells, CRC cell proliferation was significantly increased. The same phenomenon was observed in CRC xenografts in a nude mouse model, where we subcutaneously injected DENND2D-silenced or DENND2D-overexpressing HCT116 cells into the flanks of nude mice. A total of 181 stage IV colon cancer patients who had undergone surgical resection were enrolled in our study, and DENND2D-positive patients had achieved better DFS and OS than DENND2D-negative patients (DENND2D-positive group vs. DENND2D-negative group; OS: HR = 0.611, 95% CI = 0.381–0.981, *p* = 0.038; DFS: HR = 0.493, 95% CI = 0.319–0.761; *p* = 0.001). By comparing HCT116 cells expressing different levels of DENND2D mRNA by overexpression and shRNA-mediated knockdown, we observed that DENND2D suppressed the MAPK pathway. Our findings may have uncovered a novel regulatory mechanism for the DENND2D-mediated MAPK pathway in CRC.

Whether resectable CCLM should receive neoadjuvant chemotherapy remains debatable. CRS is still used in clinical practice; for patients with low risk (CRS ≤2), surgical resection is recommended. However, even low-risk patients still face a high risk of distant metastasis. In our study, of the 181 patients, 103 (56.9%) died, and 78 (43.15%) were still alive. In addition, 78 (43.1%) did not receive neoadjuvant chemotherapy, and 103 (56.8%) received neoadjuvant chemotherapy. Patients without DENND2D expression could benefit more from neoadjuvant chemotherapy. However, for patients with DENND2D expression, the benefit of neoadjuvant chemotherapy was not significant. Neoadjuvant chemotherapy could improve DENND2D-negative patient survival. DENND2D-negative patients who did not receive neoadjuvant chemotherapy experienced the worst survival.

Many oncologists have tried to identify a biomarker to identify who could benefit more from neoadjuvant chemotherapy, particularly those with low-risk CRS. MSI or MMR has been proposed as a potential biomarker. MSI-H or dMMR status has been associated with a favorable prognosis in stage II colon cancer patients. However, only a few studies have reported the relationship between the MMR status and prognosis in stage IV colon cancer [49]. A study of an Australian registry reported that MSI-H patients experienced a shorter OS than MSS patients [50]. Another study from the Mayo Clinic also reported a similar result: a shorter OS in patients with an MSI-H versus MSS status [50, 51]. However, less than 5% of stage IV colon cancer patients are MSI-H; thus, MMR status is not a good biomarker for most stage IV patients [52]. In a report based on the SEER database, KRAS mutations were found in approximately 23% of all stage IV colon cancers [52]. Of all patients who had been evaluated for KRAS mutation, no survival differences were found between KRAS-mutant patients and KRAS-WT patients [53]. Other studies reported that KRAS mutation is associated with a poor prognosis in stage IV colon cancer [54]. These discrepancies demonstrate that, although we have assumed until recently that KRAS status is a predictor for the use of EGFR inhibitors, more data are needed to support that KRAS mutation is a poor prognosis.

For the past few years, the Immunoscore^®^ has been used to help doctors and oncologists predict the prognosis of CRC patients [55]. However, all previous studies were focused on stage I to III CRC, and whether the Immunoscore^®^ could be used in stage IV colon cancer patients remains unclear [54]. In addition, the Immunoscore^®^ was also used as a predictor for chemotherapy. Chemotherapy is recommended for all stage III CC patients. We still do not have data to show the relationship between stage IV colon cancer and the response to chemotherapy. Another factor that could potentially predict the effect of adjuvant chemotherapy is circulating tumor DNA (ctDNA). Typically, ctDNA constitutes only a small proportion of total circulating free DNA [56]. An increasing number of oncologists believe ctDNA is a reliable tool that can be used as a prognostic factor in the follow-up of CRC patients because assay techniques are improving and providing better sensitivity to detect ctDNA [57]. The data related to stage IV colon cancer remain limited.

Ki-67 antigen expression is one of the most widely used markers to evaluate the proliferation of tumor cells, except for quiescent (G0 and early G1 phases) cells [58]. Whether Ki-67 expression has a relationship with the prognosis of CRC patients is unclear [59, 60]. Some studies have reported that CRC patients with high Ki-67 expression were more likely to show a poor OS [61, 62], whereas several studies have reported that high Ki-67 expression was correlated with a favorable OS [63–65]. Most studies have used a combined model including the expression of Ki-67 and other pathological parameters to predict the prognosis of CRC without distant metastasis. In our study, Ki-67 did not show a relationship with the prognosis in survival analysis. The reason is likely that the value of Ki-67 as a predictor in stage IV colon cancer was not as good as that in nonstage IV CRC.

Our data showed that stage IV colon cancer patients with DENND2D expression had better DFS and OS than those without DENND2D expression. Based on subgroup analysis, we also indicated that stage IV colon cancer patients without DENND2D expression had a worse prognosis and benefited more from neoadjuvant chemotherapy, although these results were not statistically significant. DENND2D-positive patients had a better prognosis and did not seem to benefit from neoadjuvant chemotherapy.

Based on the results of our study, DENND2D could help make decisions for stage IV colon cancer patients regarding whether they should receive neoadjuvant chemotherapy. Nine DENND2D-positive patients and 39 DENND2D-negative patients did not receive chemotherapy. Most patients in both groups (38.7% in both groups) had received XELOX as neoadjuvant chemotherapy. Based on the results of the NEW Epoc study [66], initial resectable CCLM could not benefit from EGFR inhibitor therapy. VEGF inhibitors (bevacizumab) are associated with a risk for major complications [67], and VEGF inhibitors are also not recommended by the NCCN guidelines. In our study, no patient had received targeted therapy as neoadjuvant chemotherapy. The limitations of this study are its retrospective nature and limited number of enrolled patients. More patients are needed to confirm our results. Further study is currently underway.

In summary, stage IV colon cancer patients without DENND2D expression consistently showed a worse prognosis and were more likely to benefit from neoadjuvant chemotherapy. Downregulation of DENND2D promoted CRC cell proliferation and progression in vitro and in vivo by activating the MAPK pathway. DENND2D may not only be a prognostic factor but also a predictor of sensitivity to neoadjuvant chemotherapy for stage IV colon cancer. All biopsy tissue, which was obtained by colonoscopy, should be detected for the expression of DENND2D routinely for every stage IV patient. Based on these findings, neoadjuvant chemotherapy should be strongly recommended for DENND2D-negative CCLM, even if they can achieve R0 resection for the first time. For those with DENND2D-positive expression, surgical resection could be the first choice.

## Supplementary information

Supplementary Figure 1

Supplementary Figure 2

Supplementary Figure 3

Supplementary Table

DENN blots

Supplementary Figure legends

Author Contribution Statement

Reproducibility checklist

## Data Availability

The online datasets analyzed during the current study are available in the TCGA repository (https://portal.gdc.cancer.gov/) and other experimental and clinical data are included in this article.
