# Supplementary figures and images for: Stage IV colon cancer patients without DENND2D expression benefit more from neoadjuvant chemotherapy
Source: Cell Death Dis. 2022 May 6;13(5):439. doi: 10.1038/s41419-022-04885-8 (PMC9076603; doi:10.1038/s41419-022-04885-8)

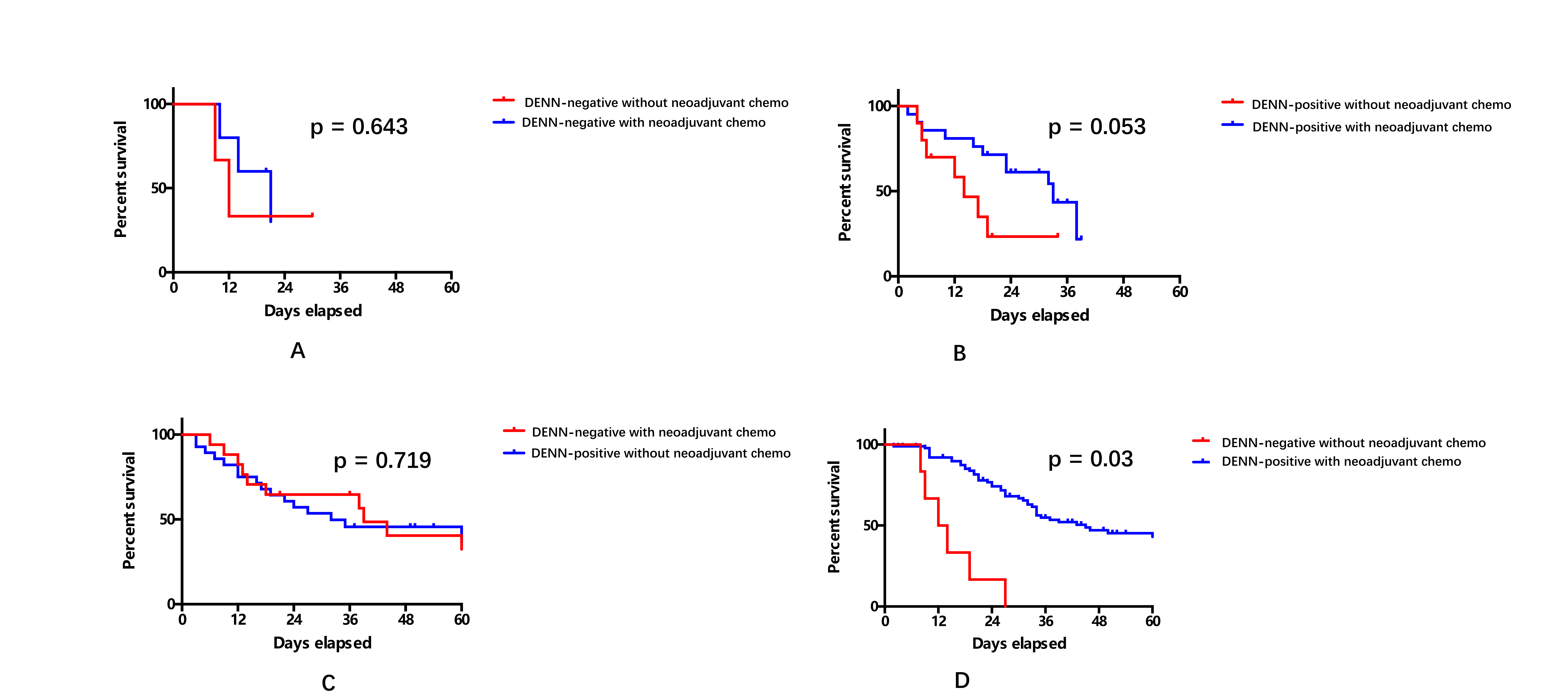

Supplement: Supplementary file 1 — Supplementary Figure 1 [file 41419_2022_4885_MOESM1_ESM.tif]

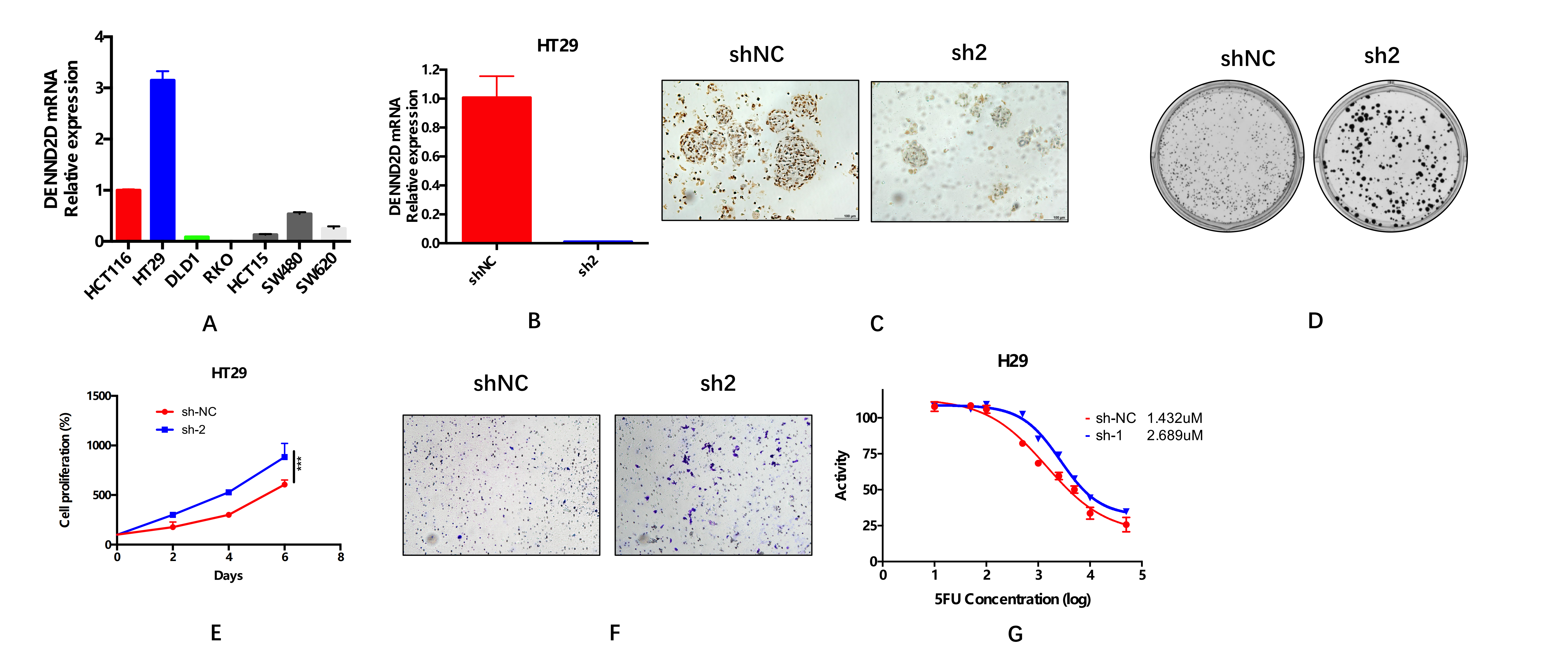

Supplement: Supplementary file 2 — Supplementary Figure 2 [file 41419_2022_4885_MOESM2_ESM.tif]

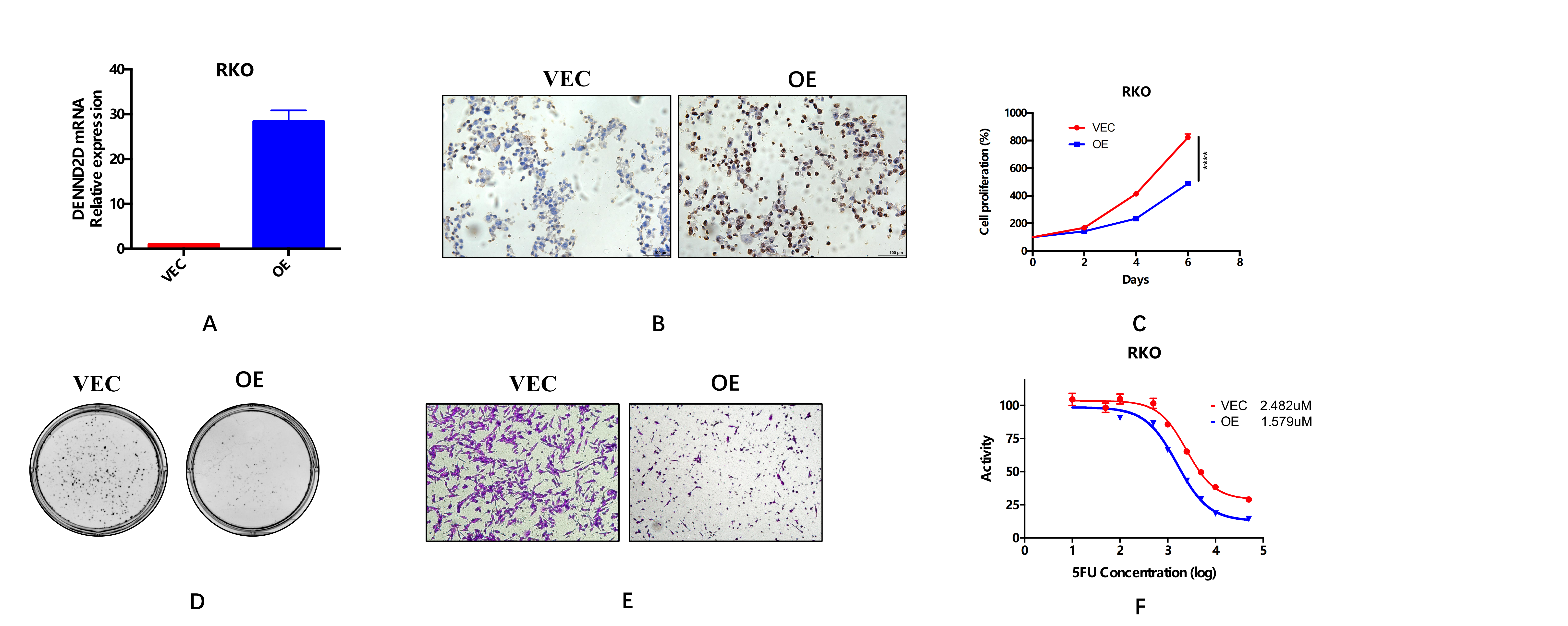

Supplement: Supplementary file 3 — Supplementary Figure 3 [file 41419_2022_4885_MOESM3_ESM.tif]
