# Supplementary material for: Stage IV colon cancer patients without DENND2D expression benefit more from neoadjuvant chemotherapy
Source: Cell Death Dis. 2022 May 6;13(5):439. doi: 10.1038/s41419-022-04885-8 (PMC9076603; doi:10.1038/s41419-022-04885-8)
Supplement: Supplementary file 4 — Supplementary Table [file 41419_2022_4885_MOESM4_ESM.docx]

Supplementary Table 1

| **Available Cancer Types** | **# Cases Shipped by BCR^*^** | **# Cases with Data^*^** | **Date Last Updated (mm/dd/yy)** |
| --- | --- | --- | --- |
| Acute Myeloid Leukemia [LAML] | 200 | 200 | 05/31/16 |
| Adrenocortical carcinoma [ACC] | 80 | 80 | 05/31/16 |
| Bladder Urothelial Carcinoma [BLCA] | 412 | 412 | 05/27/16 |
| Brain Lower Grade Glioma [LGG] | 516 | 516 | 05/02/16 |
| Breast invasive carcinoma [BRCA] | 1100 | 1097 | 05/31/16 |
| Cervical squamous cell carcinoma and endocervical adenocarcinoma [CESC] | 308 | 307 | 05/26/16 |
| Cholangiocarcinoma [CHOL] | 36 | 36 | 05/31/16 |
| Colon adenocarcinoma [COAD] | 461 | 461 | 05/27/16 |
| Esophageal carcinoma [ESCA] | 185 | 185 | 05/31/16 |
| FFPE Pilot Phase II [FPPP] | 38 | 38 | 04/28/16 |
| Glioblastoma multiforme [GBM] | 529 | 528 | 05/27/16 |
| Head and Neck squamous cell carcinoma [HNSC] | 528 | 528 | 05/03/16 |
| Kidney Chromophobe [KICH] | 66 | 66 | 06/01/16 |
| Kidney renal clear cell carcinoma [KIRC] | 536 | 536 | 05/27/16 |
| Kidney renal papillary cell carcinoma [KIRP] | 291 | 291 | 05/31/16 |
| Liver hepatocellular carcinoma [LIHC] | 377 | 377 | 06/02/16 |
| Lung adenocarcinoma [LUAD] | 521 | 521 | 06/01/16 |
| Lung squamous cell carcinoma [LUSC] | 510 | 504 | 05/26/16 |
| Lymphoid Neoplasm Diffuse Large B-cell Lymphoma [DLBC] | 48 | 48 | 05/31/16 |
| Mesothelioma [MESO] | 87 | 87 | 04/08/16 |
| Ovarian serous cystadenocarcinoma [OV] | 586 | 586 | 05/31/16 |
| Pancreatic adenocarcinoma [PAAD] | 185 | 185 | 05/06/16 |
| Pheochromocytoma and Paraganglioma [PCPG] | 179 | 179 | 05/03/16 |
| Prostate adenocarcinoma [PRAD] | 498 | 498 | 05/31/16 |
| Rectum adenocarcinoma [READ] | 172 | 171 | 06/01/16 |
| Sarcoma [SARC] | 261 | 261 | 06/01/16 |
| Skin Cutaneous Melanoma [SKCM] | 470 | 470 | 04/08/16 |
| Stomach adenocarcinoma [STAD] | 445 | 443 | 05/26/16 |
| Testicular Germ Cell Tumors [TGCT] | 150 | 150 | 06/02/16 |
| Thymoma [THYM] | 124 | 124 | 05/31/16 |
| Thyroid carcinoma [THCA] | 507 | 507 | 05/05/16 |
| Uterine Carcinosarcoma [UCS] | 57 | 57 | 04/29/16 |
| Uterine Corpus Endometrial Carcinoma [UCEC] | 548 | 548 | 06/02/16 |
| Uveal Melanoma [UVM] | 80 | 80 | 04/29/16 |
