# Supplementary material for: Stage IV colon cancer patients without DENND2D expression benefit more from neoadjuvant chemotherapy
Source: Cell Death Dis. 2022 May 6;13(5):439. doi: 10.1038/s41419-022-04885-8 (PMC9076603; doi:10.1038/s41419-022-04885-8)

Full uncut blots

Full unedited blots for:

Figure 1E DENND2D

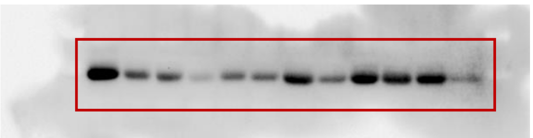

Figure1E GAPDH

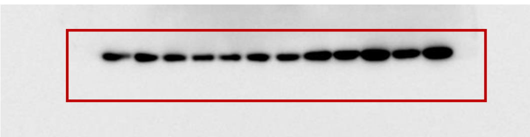

Figure3B si-NC si-1 si-2 (HCT116)

- **DENND2D**

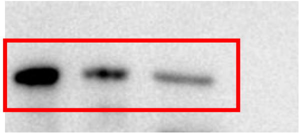

- **GAPDH**

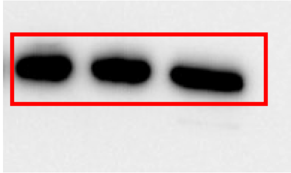

Figure3B si-NC si-1 si-2 (HT29)

- **DENND2D**

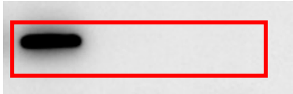

- **GAPDH**

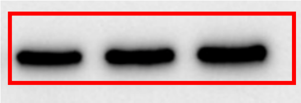

Figure3B si-NC si-1 si-2 (SW620)

- **DENND2D**

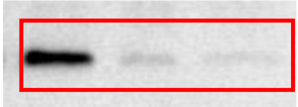

- **GAPDH**

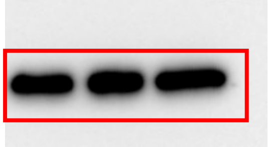

Figure 3K DENND2D（116-sh）

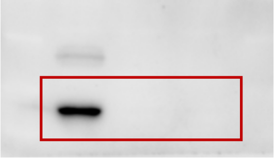

Figure 3K GAPDH（116-sh）

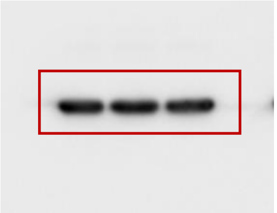

Figure 3P DENND2D (116-OE)

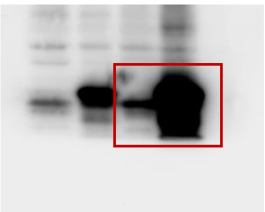

Figure 3P GAPDH (116-OE)

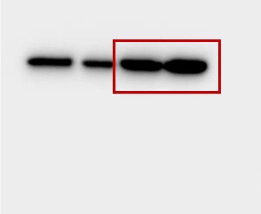

Figure5A P-p65-NF-κB

Figure5A P-38-MAPK

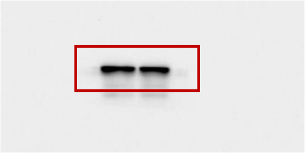

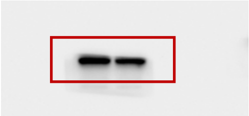

Figure5A P-SAPK/JNK

Figure5A P-MEK

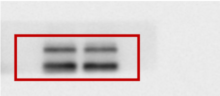

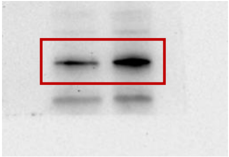

Figure5A P-AKT

Figure5A GAPDH

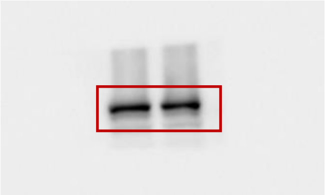

Figure 5C E-cadherin-1

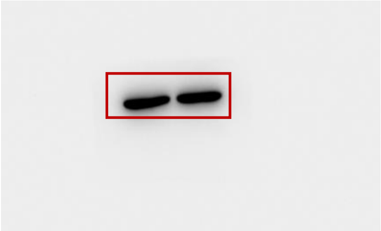

Figure 5C E-cadherin-2

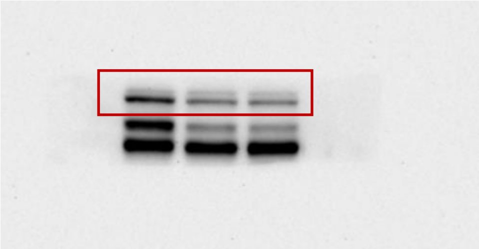

Figure 5C P-MEK1/2 S217/221-1

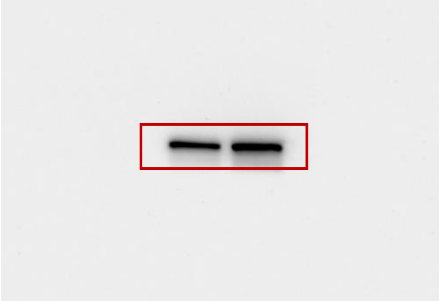

Figure 5C P-MEK1/2 S217/221-2

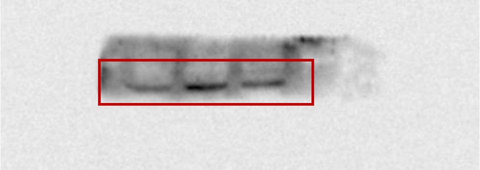

Figure 5C MEK1/2-1

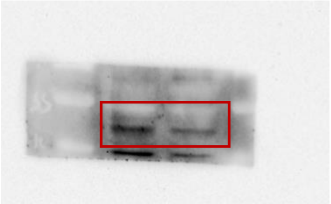

Figure 5C MEK1/2-2

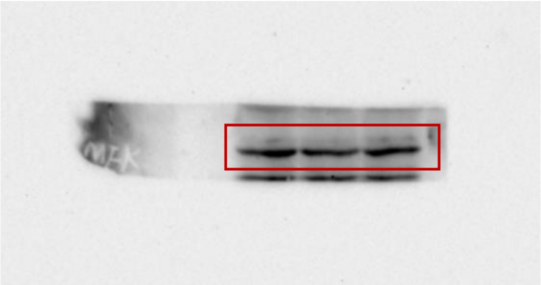

Figure 5C P-ERK1/2 T202/Y204-1

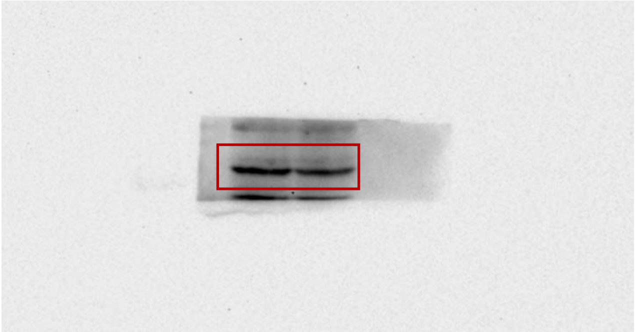

Figure 5C P-ERK1/2 T202/Y204-1

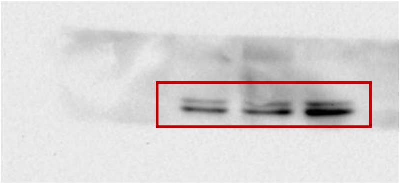

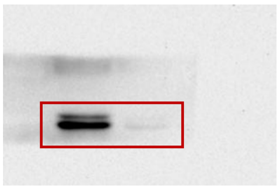

Figure 5C ERK1/2-1

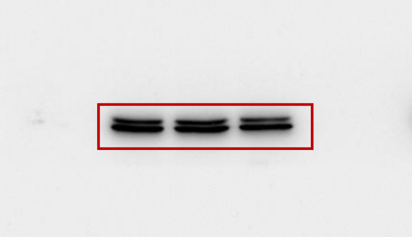

Figure 5C ERK1/2-2

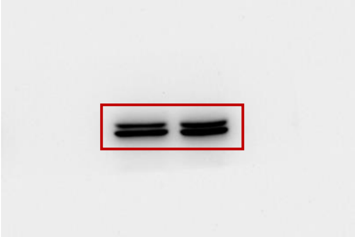

Figure 5C snail-1

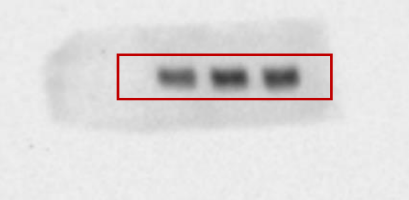

Figure 5C snail-2

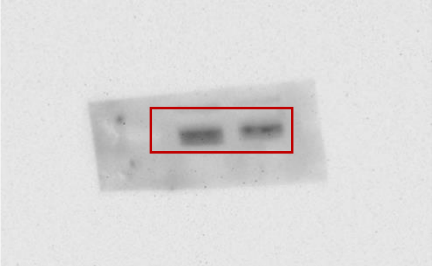

Figure 5C GAPDH-1

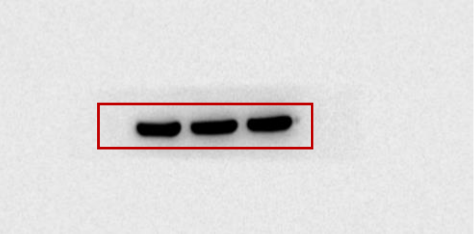

Figure 5C GAPDH-1

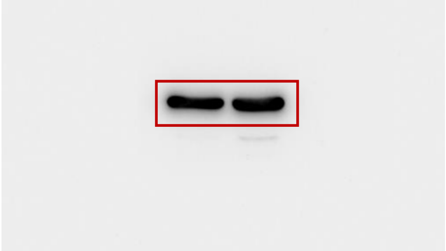

Figure 5H P-MEK1/2

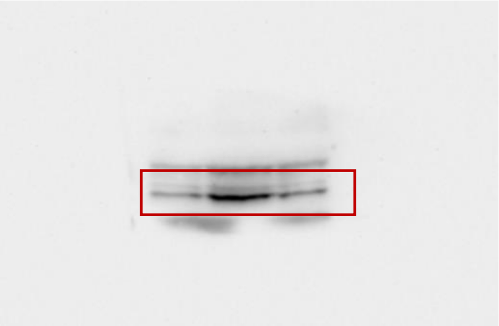

Figure 5H MEK1/2

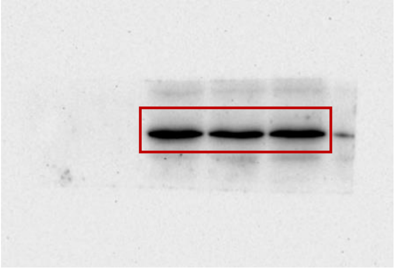

Figure 5H P-ERK1/2 T202/Y204

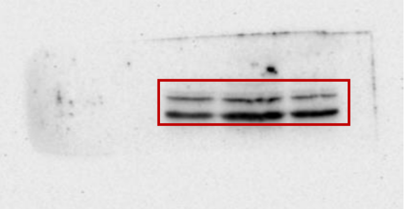

Figure 5H P-ERK1/2

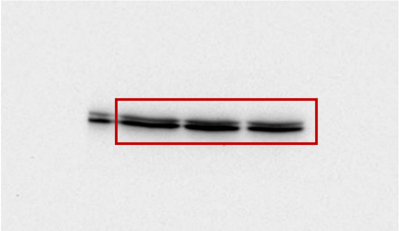

Figure 5H GAPDH

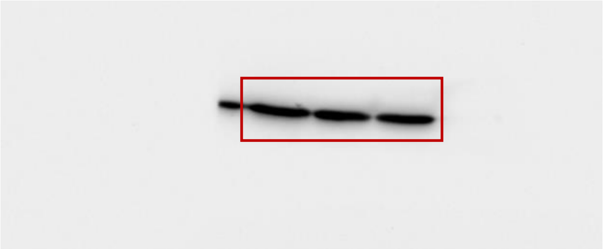

Supplement: Supplementary file 5 — DENN blots [file 41419_2022_4885_MOESM5_ESM.pdf]
