# Supplementary material for: Stage IV colon cancer patients without DENND2D expression benefit more from neoadjuvant chemotherapy
Source: Cell Death Dis. 2022 May 6;13(5):439. doi: 10.1038/s41419-022-04885-8 (PMC9076603; doi:10.1038/s41419-022-04885-8)
Supplement: Supplementary file 6 — Supplementary Figure legends [file 41419_2022_4885_MOESM6_ESM.docx]

**Supplementary Figure 1**

A. The survival rate between patients who received R1 resection with or without neoadjuvant chemotherapy in the DENND2D-negative group

B. The survival rate between patients who received R1 resection with or without neoadjuvant chemotherapy in DENND2D-positive group

C. For all R0 resected patients, the survival rate between the patients with neoadjuvant chemotherapy in DENND2D-negative group vs. the patients without neoadjuvant chemo in DENND2D-negative group

D. The survival rate between patients received R0 resection with neoadjuvant chemotherapy in DENND2D-negative group vs. the patients received R0 resection without neoadjuvant chemo in DENND2D-negative group

**Supplementary Figure 2**

A. Expression of DENND2D in CRC cell lines

B. C. DENND2D was knock down by shRNA in HT29 confirmed by WB and IHC

D. E. CRC cell proliferation was promoted by DENND2D knock down.

F. CRC cell migration was promoted by DENND2D knock down.

G. CRC cell are more sensitive to 5FU after DENND2D knock down

**Supplementary Figure 3**

A. B. DENND2D was overexpressed in RKO cell line

C. D. CRC cell proliferation was suppressed by DENND2D overexpression.

E. CRC cell migration was suppressed by DENND2D overexpression.

F. CRC cell are resistant to 5FU after DENND2D overexpression
