# Supplementary material for: Stage IV colon cancer patients without DENND2D expression benefit more from neoadjuvant chemotherapy
Source: Cell Death Dis. 2022 May 6;13(5):439. doi: 10.1038/s41419-022-04885-8 (PMC9076603; doi:10.1038/s41419-022-04885-8)
Supplement: Supplementary file 7 — Author Contribution Statement [file 41419_2022_4885_MOESM7_ESM.docx]

**Author Contributions**

All authors helped to perform the study. Wen-juan Ma and Yukun Chen wrote the manuscript and performed the procedures and data analysis. J.H. Peng, Chaoming Tang, Ling Zhang and Min Liu performed the experiments and data analysis. Z.Z. Pan wrote the manuscript and analyzed the data. G. Chen collected the data and design the study. H.N. Xu, Shanshan Hu and Hua Tan analyzed the data and designed the study. Z.G. Zhou wrote the manuscript and conceived the study. Rong-xin Zhang designed the study and analyzed the data.
