# Supplementary material for: Stage IV colon cancer patients without DENND2D expression benefit more from neoadjuvant chemotherapy
Source: Cell Death Dis. 2022 May 6;13(5):439. doi: 10.1038/s41419-022-04885-8 (PMC9076603; doi:10.1038/s41419-022-04885-8)
Supplement: Supplementary file 8 — Reproducibility checklist [file 41419_2022_4885_MOESM8_ESM.pdf]

Corresponding Author Name: Rongxin Zhang
Manuscript Number: JDIS-21-3981RR

SPRINGER NATURE

Reporting Summary

Springer Nature wishes to improve the reproducibility of the work that we publish. This checklist is used to ensure good reporting standards and to improve the reproducibility. Please respond completely to all questions relevant to your manuscript. For more information, please read the journal's Guide to Authors.

☑ **Check here to confirm that the following information is available in the Material & Methods section:**

- **The exact sample size (*n*)** for each experimental group/condition, given as a number, not a range
- **A description of the sample collection** allowing the reader to understand whether the samples represent technical or biological replicates (including how many animals, litters, culture, etc.)
- **A statement of how many times the experiment shown was replicated in the laboratory**
- **Definitions of statistical methods and measures**: For small sample sizes (n<5) descriptive statistics are not appropriate, instead plot individual data points
  - Very common tests, such as *t*-test, simple χ^2 tests, Wilcoxon and Mann-Whitney tests, can be unambiguously identified by name only, but more complex techniques should be described in the methods section
  - Are tests one-sided or two-sided?
  - Are there adjustments for multiple comparisons?
  - **Statistical test results**, e.g., ***P* values**
  - Definition of **'center values'** as **median or mean**;
  - Definition of **error bars as s.d. or s.e.m. or c.i.**

Please ensure that the answers to the following questions are reported in the manuscript itself. We encourage you to include a specific subsection in the methods section for statistics, reagents and animal models. Below, provide the page number or section and paragraph number.

Statistics and general methods

	Reported in section/paragraph or page #
1. How was the sample size chosen to ensure adequate power to detect a pre-specified effect size? (Give section/paragraph or page #)	Page 7, line 131-132, "One hundred eighty-one samples wer e obtained from patients at Sun Yat-Sen University Cancer C enter from May 1, 2003, to May 1, 2016. "
For animal studies, include a statement about sample size estimate even if no statistical methods were used.	Page 21, line 416-418, "DENND2D-silenced or DENND2D-ov erexpressing HCT116 cells were subcutaneously injected int o the flanks of nude mice to establish a xenograft model (5 mice in each group)."
2. Describe inclusion/exclusion criteria if samples or animals were excluded from the analysis. Were the criteria pre-established? (Give section/paragraph or page #)	Page 7, line 133-136, "All patients were diagnosed with initi ally resectable CRLM and received R0 or R1 surgical resectio n. All the patients were pathologically confirmed to have co lon adenocarcinoma."
3. If a method of randomization was used to determine how samples/animals were allocated to experimental groups and processed, describe it. (Give section/paragraph or page #)	n/a
For animal studies, include a statement about randomization even if no randomization was used.	Page 12, line 235-237," Female BALB/c nude mice (4–5 wee ks; 15–18 g; SLRC Laboratory Animal Co., Shanghai, China) were divided into five groups randomly"

4. If the investigator was blinded to the group allocation during the experiment and/or when assessing the outcome, state the extent of blinding. (Give section/paragraph or page #)	n/a
For animal studies, include a statement about blinding even if no blinding was done.	n/a
5. For every figure, are statistical tests justified as appropriate?	Yes, Page 15, line 290-294, "The clinical and follow-up data were analyzed using SPSS v19.0 and R language. χ2, continuity correction χ2, and Fisher's exact tests were used to asses
Do the data meet the assumptions of the tests (e.g., normal distribution)?	Yes, Page 15, line 294-295, " The variation of each group had been estimated properly and met the basic assumptions of the tests."
Is there an estimate of variation within each group of data?	Page 15, line 294-295, "The variation of each group had been estimated properly and met the basic assumptions of the tests."
Is the variance similar between the groups that are being statistically compared? (Give section/paragraph or page #)	Yes, Page 15, line 294-295, "The variation of each group had been estimated properly and met the basic assumptions of the tests."

Reagents

Reported in section/paragraph or page #

6. Report the source of antibodies (vendor and catalog number)	Page 10, line 193-195, "DENND2N (ab184799; Abcam); GAPDH (D16H11), phosphor-MEK1/2 (Ser221) (166F8), MEK1/2 (L38C12), phosphor-ERK1/2 (Thr202/Tyr204) (#9101), and E
7. Identify the source of cell lines and report if they were recently authenticated (e.g., by STR profiling) and tested for mycoplasma contamination	Page 8, line 143-153, "HCT116, HT29 and SW620 CRC cell lines were purchased from American Type Culture Collection (ATCC, Manassas, VA, USA) and cultured according to ATCC guidelines. HCT116 and HT29 cells were cultivated in McCoy's 5A medium (KGM4892N; KeyGEN BioTECH), and SW620 ce

Animal Models

Reported in section/paragraph or page #

8. Report species, strain, sex and age of animals	Page 12, line 235-237, "Female BALB/c nude mice (4–5 weeks; 15–18 g; SLRC Laboratory Animal Co., Shanghai, China)"
9. For experiments involving live vertebrates, include a statement of compliance with ethical regulations and identify the committee(s) approving the experiments.	Page 12, line 233-235, "The animal experiments were approved by the SYSUCC Institutional Animal Care and Usage Committee in accordance with the Animal Welfare and Rights in China."

10. We recommend consulting the ARRIVE guidelines (*PLoS Biol.* **8**(6), e1000412,2010) to ensure that other relevant aspects of animal studies are adequately reported.

Human subjects

	Reported in section/paragraph or page #
11. Identify the committee(s) approving the study protocol.	Page 7-8, line 137-140, "Studies involving human tissue sampl es were carried out in accordance with the guidelines approve d by the Ethics Committees of Sun Yat-Sen University Cancer C [illegible]
12. Include a statement confirming that informed consent was obtained from all subjects.	Page 7, line 132-133, "Informed consent was obtained from al l the patients before tissue collection."
13. For publication of patient photos, include a statement confirming that consent to publish was obtained.	n/a
14. Report the clinical trial registration number (at ClinicalTrials.gov or equivalent).	n/a

15. For phase II and III randomized controlled trials, please refer to the CONSORT statement and submit the CONSORT checklist with your submission.

16. For tumor marker prognostic studies, we recommend that you follow the REMARK reporting guidelines.

Data deposition

	Reported in section/paragraph or page #
17. Provide accession codes for deposited data. Data deposition in a public repository is mandatory for:<br>a. Protein, DNA and RNA sequences<br>b. Macromolecular structures<br>c. Crystallographic data for small molecules<br>d. Microarray data	Page 14, line 281-284, "Gene expression data of each cancer were downloaded from the TCGA data portal (https://portal.g dc.cancer.gov/) (Supplemental Table 1). A total of 20531 gene s (protein coding and noncoding) were included in the TCGA Il lumina HiSeq RNASeq V2 data."<br>Page 15, line 299-300, "All data in our study have been record ed at Sun Yat-sen University Cancer Center for future referenc e (Number RDDB2021001606)."

Deposition is strongly recommended for many other datasets for which structured public repositories exist; more details on our data policy are available in the Guide to Authors. We encourage the provision of other source data in supplementary information or in unstructured repositories such as Figshare and Dryad. We encourage publication of Data Descriptors (see Scientific Data) to maximize data reuse.

18. If computer code was used to generate results that are central to the paper's conclusions, include a statement in the Methods section under "**Code availability**" to indicate whether and how the code can be accessed. Include version information as necessary and any restrictions on availability.	Page 15, line 287-288, "Gene expression was visualized with b ox plots by the R package ggplot2, version 3.3.5."
